# Intravenous Ketamine or Esketamine in Adolescents With Major Depressive Disorder: A Systematic Review

**DOI:** 10.31083/AP48866

**Published:** 2026-08-27

**Authors:** Zhen-Juan Qin, Xiao-Ming Li, Jia-Bao Li, Zhi Li, Xin Wei, Hui-Lin Hu, Yu-Jiao Chen, Su-Rong Xu, Zhi-Yuan Xie, Xin-Hu Yang, Wen-Juan Dai, Yu-Hua Wei

**Affiliations:** ^1^Department of Psychiatry, The Brain Hospital of Guangxi Zhuang Autonomous Region, 545005‌ Liuzhou, Guangxi, China; ^2^School of Clinical Medicine, School of Graduate Studies, Youjiang Medical University for Nationalities, 533000 Baise, Guangxi, China; ^3^Department of Psychiatry, The Affiliated Brain Hospital, Guangzhou Medical University, 510370 Guangzhou, Guangdong, China; ^4^Key Laboratory of Neurogenetics and Channelopathies of Guangdong Province and the Ministry of Education of China, Guangzhou Medical University, 510260 Guangzhou, Guangdong, China; ^5^Department of Medical Administration, Liuzhou Health Commission, 545001 Liuzhou, Guangxi, China

**Keywords:** ketamine, esketamine, major depressive disorder, adolescents, systematic review

## Abstract

**Background:**

The therapeutic efficacy and safety of intravenous ketamine or esketamine in adolescents with major depressive disorder (MDD) remain insufficiently defined. This systematic review of randomized controlled trials (RCTs) aimed to evaluate the efficacy, safety, and tolerability of intravenous ketamine or esketamine in this population.

**Methods:**

A comprehensive search of four international databases (PubMed, EMBASE, PsycINFO, and the Cochrane Library) was conducted to identify RCTs comparing intravenous ketamine or esketamine with intravenous midazolam in adolescents with MDD.

**Results:**

Three RCTs involving a total of 134 adolescents with MDD were included. Two trials assessed the antidepressant effects of intravenous ketamine compared with intravenous midazolam, with inconsistent findings. No significant advantage of intravenous ketamine over intravenous midazolam was observed in reducing anxiety symptoms (1 RCT). One trial compared intravenous esketamine with intravenous midazolam and evaluated antidepressant, anti-suicidal, and neurocognitive outcomes, demonstrating the superiority of intravenous esketamine. Both intravenous ketamine and intravenous esketamine were associated with significantly higher rates of dissociative symptoms (1 RCT each), and intravenous esketamine was also linked to a higher incidence of psychopathological symptoms (1 RCT). Systematic reporting of adverse events was provided in only one of the three included RCTs.

**Conclusions:**

Given the limited number of studies, small sample sizes, and methodological heterogeneity of the available RCTs, the current evidence base remains preliminary and insufficient to support definitive conclusions regarding the efficacy and safety of intravenous ketamine/esketamine in adolescents with MDD. These findings highlight the need for further adequately powered RCTs to establish robust and clinically meaningful evidence.

## Main Points

1. Available trial data are insufficient to determine the efficacy of intravenous ketamine/esketamine for depression, anxiety, suicidal ideation, or neurocognitive deficits in adolescents with major depressive disorder (MDD).

2. Both intravenous ketamine and esketamine were associated with markedly higher rates of dissociative symptoms compared to midazolam.

3. Only one out of three randomized controlled trials (RCTs) systematically reported adverse events.

## 1. Introduction

Elevated depressive symptoms are common among adolescents, with prevalence estimates approaching 30%–35%, whereas the prevalence of major depressive disorder (MDD) is substantially lower, at approximately 4%–8% [1]. Adolescent depression is also frequently associated with comorbid psychiatric symptoms [2]. MDD in adolescents leads to significant impairments across multiple domains, including familial, academic, and interpersonal functioning [3]. Furthermore, adolescent depression is associated with an increased risk of suicidal behavior [4].

Antidepressants, psychotherapy, and non-invasive brain stimulation (NIBS) can be used for the acute management of MDD in adolescents [5,6]. However, the risk-benefit profile of antidepressants, particularly selective serotonin reuptake inhibitors, does not clearly favor their use in acute treatment of adolescent MDD [7]. While cognitive behavioral therapy (CBT) represents the gold standard in evidence-based psychotherapy, and combining it with pharmacotherapy can lead to faster and more substantial clinical improvement in the acute phase [8], its effectiveness is limited by high dropout rates, with more severe baseline depression being a predictor of earlier discontinuation [9]. Repetitive transcranial magnetic stimulation (rTMS) is the most widely employed NIBS method for adolescent depression. However, the optimal stimulation dose, site, and parameters for adolescents with MDD remain undefined, likely differing from those established for adult depression [10]. Therefore, an urgent requirement exists to identify innovative therapeutic approaches for the expedited treatment of adolescent depression.

Ketamine and esketamine are closely related compounds that induce a range of transcriptional changes, engaging neuroplastic, inflammatory, and metabolic pathways [11]. Ketamine, an N-methyl-D-aspartate (NMDA) receptor antagonist, consists of two enantiomers, S-ketamine and R-ketamine, and has received Food and Drug Administration (FDA) approval for application as a sedative, analgesic, and general anesthetic [12]. Esketamine, representing the S-enantiomer of the ketamine racemate, demonstrates greater affinity for the NMDA receptor compared to the R-enantiomer and has obtained FDA approval for treating treatment-resistant depression (TRD) in adult patients. Although the primary molecular target of ketamine necessary for its antidepressant activity is still under debate, the blockade of the NMDA receptor is the best-studied possible explanatory hypothesis of the antidepressant effects of ketamine [13]. Additionally, mechanistic target of rapamycin plays a role in the antidepressant effects of esketamine [14]. Clinically, both single and repeated intravenous infusions of sub-anesthetic ketamine or esketamine have demonstrated rapid antidepressant effects in adult depression [15,16,17,18,19].

Accumulating data indicate that intravenous ketamine/esketamine may demonstrate both safety and effectiveness in managing adolescent depression [20,21]. Nonetheless, comprehensive data regarding the therapeutic efficacy, safety, and tolerability of these interventions across adolescent populations remain limited. Several systematic reviews [5,22,23,24] incorporating non-randomized control studies have found that ketamine/esketamine can reduce depressive symptoms and exhibit anti-suicidal properties among adolescent populations presenting with TRD. For example, Bruton et al.’s review [22], which included seven case studies, six observational studies, three randomized trials, and six secondary data analyses, concluded that ketamine is safe and may benefit mood disorders, anxiety, and suicidality in youth. However, the predominance of case reports and observational studies, which lacked randomization and blinding, raises concerns about the potential influence of placebo effects or other biases. Additionally, the administration routes of ketamine/esketamine varied (intranasal, intravenous, intramuscular), and the differential effects of these formulations and delivery methods remain underexplored. Midazolam served as an active placebo given its comparable pharmacokinetic characteristics, functioning as a reasonable comparator for the nonspecific behavioral effects of ketamine [21]. To further elucidate the role of intravenous ketamine/esketamine in adolescent depression and provide a more robust foundation for clinical applications, this systematic review exclusively focused on randomized controlled trials (RCTs) to assess the efficacy and safety of intravenous ketamine or esketamine versus intravenous midazolam in adolescents with MDD.

## 2. Methods

This review was conducted in accordance with the Preferred Reporting Items for Systematic Reviews and Meta-Analyses (PRISMA) 2020 Guidelines (See **Supplementary Material**).

### 2.1 Search Strategy

A systematic search was executed in four international databases (PsycINFO, PubMed, EMBASE, and Cochrane Library) from their inception through June 2025 by two researchers. The search terms utilized in PubMed comprised: (“Ketamine”[Mesh] OR Ketamine OR Esketamine OR s-ketamine OR arketamine OR r-ketamine OR Spravato OR NMDA-receptor antagonist OR NMDA receptor antagonist OR NMDA antagonist OR NMDAR antagonist OR racemic-ketamine OR “ketalar” OR N-methyl-D-aspartate OR esketamine nasal spray) AND (“depression”[MeSH] OR depress* OR dysphor* OR melanchol* OR antidepress*) AND (adolesc* OR young adult OR youth OR teen* OR young people OR young person*) AND (random* OR sham OR placebo OR control). Supplementary articles were obtained through manual examination of reference lists from the included studies [20,21,25], as well as relevant reviews [5,22,23,24].

### 2.2 Selection Criteria

Per the Preferred Reporting Items for Systematic Reviews and Meta-Analyses (PRISMA) guidelines [26], selection parameters followed the ***PICOS*** (Population, Intervention, Comparison, Outcomes, and Study design) framework: Participants (***P***): Adolescent patients (12–18 years) with MDD diagnosis based on the diagnostic criteria established in each included study. Intervention (***I***) versus Comparison (***C***): Intravenous ketamine or esketamine combined with treatment as usual (TAU) versus intravenous midazolam plus TAU, or intravenous ketamine or esketamine monotherapy versus intravenous midazolam monotherapy or intravenous midazolam plus TAU. Outcomes (***O***): The primary outcome encompassed alterations in depressive symptoms, measured through validated instruments like the Children’s Depression Inventory (CDI) [27], the Children’s Depression Rating Scale–Revised (CDRS-R) [28], or the Montgomery-Åsberg Depression Rating Scale (MADRS) [29]. Key secondary outcomes encompassed: (1) changes in suicidal ideation assessed through standardized scales (e.g., the Suicide Ideation and Behavior Assessment Tool (SIBAT) [30]), (2) anxiety symptoms evaluated using standardized scales (e.g., the Hamilton Anxiety Rating Scale (HAMA) [31]), (3) neurocognitive function evaluated through standardized scales (e.g., the MATRICS Consensus Cognitive Battery (MCCB) [32,33]), (4) discontinuation rate, and (5) adverse events. Study Design (***S***): Only published RCTs in English comparing intravenous ketamine or esketamine to intravenous midazolam in adolescents with MDD were eligible for inclusion. RCTs focused on ketamine for adult MDD [34] and esketamine nasal spray [35] were excluded. Additionally, case reports/series, animal trials, and review articles were excluded. Among multiple published articles derived from the same dataset [21,36,37]; [20,38,39], only those with the most comprehensive data [20,21] were included.

### 2.3 Data Extraction

Two researchers independently retrieved and confirmed data from each included RCT. Disagreements were addressed through consultation with a senior author. Authors were contacted to clarify information when necessary.

### 2.4 Study Quality Assessment

The identical pair of researchers independently evaluated the quality of each RCT utilizing both the Cochrane Risk of Bias tool [40] and the Jadad scale (0–5 points) [41]. RCTs achieving a Jadad score of 3 or above were categorized as “high quality” [42].

## 3. Results

### 3.1 Study Selection

As illustrated in Fig. 1, 882 unduplicated studies were ascertained, encompassing one manually retrieved [25]. After screening titles, abstracts, and full texts, three RCTs [20,21,25] were included in this systematic review.

**Fig. 1.**
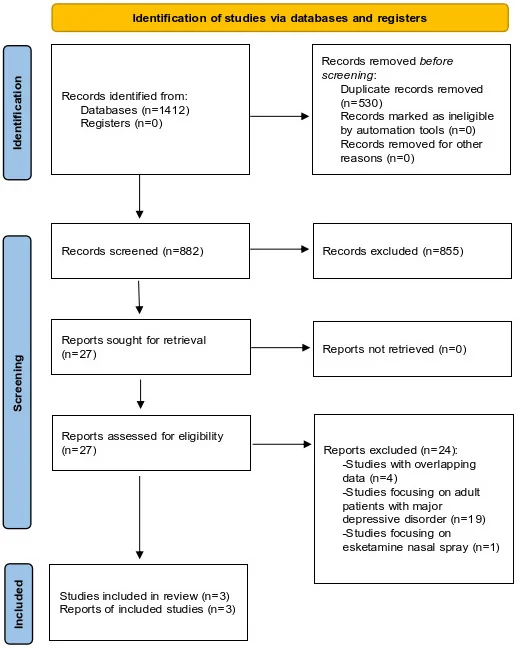
**PRISMA flow diagram**. PRISMA, Preferred Reporting Items for Systematic Reviews and Meta-Analyses.

### 3.2 Study Characteristics

Table 1 (Ref. [20,21,25,36]) presents the demographic and clinical characteristics of all included studies. The three RCTs, published between 2021 and 2024, involved 134 participants. Two studies (n = 80) [21,25] administered intravenous ketamine (0.5 mg/kg), while the remaining study (n = 54) [20] used intravenous esketamine (0.25 mg/kg). Two included RCTs [20,21] allowed the use of antidepressants, antipsychotics, and mood stabilizers, while Macejova et al. [25] required stopping pre-existing antidepressants during the infusion phase. The participants had a weighted mean age of 15.0 years, and 92.5% were female. The total number of treatment sessions ranged from 1 to 6.

**Table 1.** **Patient characteristics and intravenous ketamine/esketamine or midazolam parameters of each included study**.

Studies (country)	N^a^	Design: -Blinding -Setting (%)	-Diagnostic criteria -Diagnosis (TRD %)	-Illness duration: years -Male (%) -Age: years (range)	-Ketamine/esketamine groups (N) -Midazolam groups (N)	-Dose (mg/kg) -Number of sessions (n/week)		Jadad score
						Ketamine/esketamine groups	Midazolam groups	
Dwyer et al., 2021 [21] (USA)	17	-DB -Outpatients^b^ (100)	-DSM-5 -MDD (100)	-2.5 -4 (23.5) -15.5 (13–17)	-Ketamine + TAU (n = 6) -Midazolam + TAU (n = 11)	-0.500 -1^c^	-0.045 -1^c^	4
Macejova et al., 2024 [25] (Slovakia)	63	-DB -Inpatients (100)	-DSM-5 -MDD (100)	-NR -0 (0) -15.1^d^ (12–18)	-Ketamine monotherapy (n = 35) -Midazolam monotherapy (n = 28)	-0.500 -6 (3)	-0.045 -6 (3)	4
Zhou et al., 2023 [20] (China)	54	-DB -Inpatients (100)	-DSM-5 -MDD (NR)	-1.8 -6 (11.1) -14.7 (13–18)	-Esketamine + TAU (n = 27) -Midazolam + TAU (n = 27)	-0.250 -3 (3)	-0.020 -3 (3)	5

^a^ The data were extracted based on random assignment. ^b^ The data originate from another article based on the same experiment (see Reference [36]). ^c^ Participants receiving a single infusion. ^d^ Available data were extracted based on the mean baseline value of observed cases. Abbreviations: DB, double blind; DSM-5, Diagnostic and Statistical Manual of Mental Disorders 5th edition; MDD, major depressive disorder; N, number of patients; NR, not reported; TAU, treatment as usual; TRD, treatment-resistant depression.

### 3.3 Assessment of Study Quality

The included RCTs received Jadad scores between 4 and 5, indicating high quality (Jadad score ≥3) for all studies [20,21,25]. Based on the Cochrane risk of bias evaluation (Fig. 2), all studies (3/3, 100%) exhibited a low risk of bias regarding participant and personnel blinding, outcome assessment blinding, and incomplete outcome data. A single study (1/3, 33.3%) displayed a low risk of bias regarding random sequence generation and allocation concealment. One study (1/3, 33.3%) was judged to have a high risk of selective reporting bias, as the primary outcome was changed in the trial registry.

**Fig. 2.**
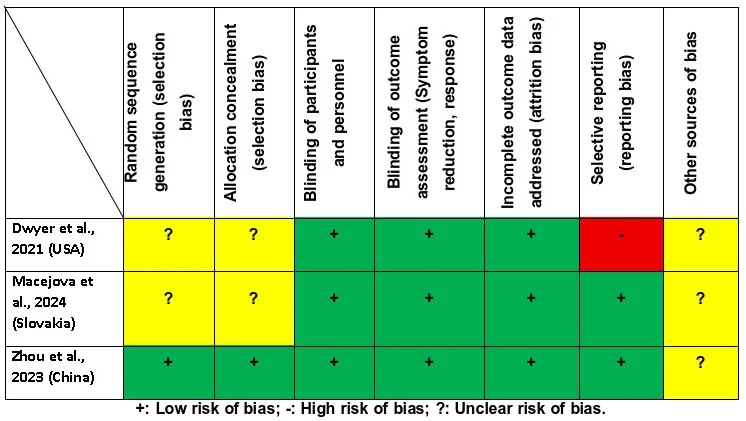
**Cochrane risk of bias**.

### 3.4 Changes in Depressive Symptoms

#### 3.4.1 Intravenous Ketamine Versus Midazolam

As outlined in Table 2 (Ref. [20,21,25]), two RCTs (2/2, 100%) [21,25] evaluated the antidepressant effectiveness of intravenous ketamine compared to intravenous midazolam in adolescents with MDD, producing conflicting findings. Dwyer et al. [21] demonstrated a notable decrease in depressive symptoms with intravenous ketamine relative to intravenous midazolam, as determined by the MADRS. Conversely, Macejova et al. [25] observed no marked distinction between the two groups.

**Table 2.** **Intravenous ketamine and esketamine in adolescents with MDD: clinical efficacy**.

Studies	Assessment scales	Findings
Intravenous ketamine versus midazolam		
The severity of depressive symptoms		
Dwyer et al., 2021 [21] (USA)	CDRS-R and MADRS	Intravenous ketamine significantly improved depressive symptoms as measured by the MADRS compared to midazolam in raw score analyses, but not the CDRS-R.
Macejova et al., 2024 [25] (Slovakia)	CDI and MADRS	No significant group differences were found regarding depressive symptoms as measured by the CDI and MADRS.
The severity of anxiety symptoms		
Dwyer et al., 2021 [21] (USA)	NR	NR
Macejova et al., 2024 [25] (Slovakia)	HAMA	No significant group differences were found regarding anxiety symptoms as measured by the HAMA.
The severity of suicidal ideation		
Dwyer et al., 2021 [21] (USA)	NR	NR
Macejova et al., 2024 [25] (Slovakia)	NR	NR
Intravenous esketamine versus midazolam		
The severity of depressive symptoms		
Zhou et al., 2023 [20] (China)	MADRS	Intravenous esketamine significantly improved depressive symptoms as measured by the MADRS compared to midazolam at day 6, but not days 2 or 4.
The severity of anxiety symptoms		
Zhou et al., 2023 [20] (China)	NR	NR
The severity of suicidal ideation		
Zhou et al., 2023 [20] (China)	C-SSRS and SSI-5	Esketamine significantly improved suicidal ideation as measured by the C-SSRS and the SSI-5 compared to midazolam.

Abbreviations: CDI, Children’s Depression Inventory; CDRS-R, Children’s Depression Rating Scale–Revised; C-SSRS, Columbia Suicide Severity Rating Scale; HAMA, Hamilton Anxiety Rating Scale; MADRS, Montgomery-Åsberg Depression Rating Scale; SSI-5, The first 5 items of Beck Scale for Suicide Ideation.

#### 3.4.2 Intravenous Esketamine Versus Midazolam

A single RCT (1/1, 100%) [20] examined the antidepressant efficacy of intravenous esketamine versus intravenous midazolam in adolescents with MDD, showing notable amelioration in depressive symptoms as evaluated by the MADRS on day 6, though not on days 2 or 4.

### 3.5 Changes in Anxiety Symptoms

#### 3.5.1 Intravenous Ketamine Versus Midazolam

One RCT (1/2, 50%) [25] evaluated the anxiolytic properties of intravenous ketamine versus intravenous midazolam in adolescents with MDD, demonstrating no statistically significant differences (Table 2).

#### 3.5.2 Intravenous Esketamine Versus Midazolam

No RCT evaluated the anxiolytic efficacy of intravenous esketamine versus intravenous midazolam in adolescents with MDD.

### 3.6 Changes in Suicidal Ideation

#### 3.6.1 Intravenous Ketamine Versus Midazolam

No RCT examined the anti-suicidal efficacy of intravenous ketamine versus intravenous midazolam in adolescents with MDD.

#### 3.6.2 Intravenous Esketamine Versus Midazolam

A single RCT (1/1, 100%) [20] examined the anti-suicidal efficacy of intravenous esketamine among adolescents diagnosed with MDD, demonstrating that intravenous esketamine was markedly more effective than midazolam in reducing suicidal ideation, as assessed by the Columbia Suicide Severity Rating Scale (C-SSRS) and The first 5 items of Beck Scale for Suicide Ideation (SSI-5) (Table 2).

### 3.7 Neurocognitive Function

#### 3.7.1 Intravenous Ketamine Versus Midazolam

No RCT examined the neurocognitive function of intravenous ketamine versus intravenous midazolam in adolescents with MDD.

#### 3.7.2 Intravenous Esketamine Versus Midazolam

A single RCT (1/1, 100%) [20] reported that intravenous esketamine markedly improved processing speed compared to midazolam in adolescents with MDD, as measured by the MCCB, with no notable distinctions in other neurocognitive domains (Table 3) (Ref. [20,21,25,39]).

**Table 3.** **Intravenous ketamine and esketamine in adolescents with MDD: neurocognitive function**.

Study	Assessment scales	Findings
Intravenous ketamine versus midazolam		
Dwyer et al., 2021 [21] (USA)	NR	NR
Macejova et al., 2024 [25] (Slovakia)	NR	NR
Intravenous esketamine versus midazolam		
Zhou et al., 2023 [20] (China)^a^	MCCB	Esketamine significantly improved processing speed as measured by the MCCB compared to midazolam, with no significant differences in other neurocognitive domains.

^a^ The data originate from another article based on the same experiment (see Reference [39]). Abbreviations: MCCB, MATRICS Consensus Cognitive Battery.

### 3.8 Adverse Events and Discontinuation Rate

**Supplementary Table 1** summarizes the dissociative and psychopathological symptoms reported across the three RCTs [20,21,25]. Two RCTs (2/3, 66.7%) [20,21] comparing intravenous ketamine/esketamine with intravenous midazolam in adolescents with MDD found a higher incidence of dissociative symptoms within 30–60 minutes post-infusion in the intravenous ketamine/esketamine groups. One RCT [20] evaluated the psychopathological symptoms of intravenous esketamine versus intravenous midazolam in adolescents with MDD, reporting more psychopathological symptoms on days 1 and 3.

The adverse events documented in the three RCTs [20,21,25] are depicted in **Supplementary Table 2**. One RCT [20] evaluated adverse events following intravenous esketamine versus intravenous midazolam in adolescents with MDD, reporting higher incidences of nausea and dissociation with intravenous esketamine.

Discontinuation rates ranged from 0% to 22.9% in the intravenous ketamine/esketamine group and from 0% to 11.1% in the intravenous midazolam group across all included RCTs [20,21,25] (**Supplementary Table 2**).

## 4. Discussion

This systematic review encompassed three RCTs comprising 134 adolescent patients diagnosed with MDD to examine the efficacy and safety of intravenous ketamine/esketamine versus midazolam. The primary observations are as follows: (1) Insufficient evidence exists to determine whether intravenous ketamine/esketamine is superior to intravenous midazolam in alleviating suicidal ideation, anxiety, depressive symptoms, or neurocognitive deficits in adolescents with MDD; (2) Intravenous ketamine/esketamine was associated with adverse dissociative and psychopathological symptoms; (3) Only one of the three RCTs, which focused on intravenous esketamine versus intravenous midazolam, systematically reported adverse events.

The efficacy of intravenous ketamine/esketamine versus intravenous midazolam for reducing depressive symptoms in adolescents with MDD continues to be unclear. One RCT (1/2, 50%) [21] reported mixed results, while another (1/2, 50%) [25] demonstrated no notable distinction between intravenous ketamine and intravenous midazolam in improving depressive symptoms. A single RCT (1/1, 100%) [20] demonstrated a marked improvement in depressive symptoms with intravenous esketamine versus intravenous midazolam on day 6, but not on days 2 or 4. Notably, evidence outside of intravenous administration also exists. For example, intranasal esketamine in conjunction with a comprehensive standard of care has been shown to rapidly improve depressive symptoms among adolescents with MDD at imminent risk for suicide [35]. These results are likely influenced by the confounding effect of concurrent antidepressant use, the potent psychoactive placebo effect of intravenous midazolam in adolescents with MDD, and potential gender-specific differences in treatment response. The synergistic impact of combining ketamine/esketamine with antidepressants is well-documented, with studies showing that such combinations not only substantially alleviate symptoms [20] but also help sustain therapeutic efficacy and reduce recurrence risk [43]. This suggests the potential necessity of integrating antidepressants into combination treatment regimens. Additionally, midazolam, as an active placebo, has significant psychoactive effects that can influence patients’ overall perception of mood improvement [25]. This is especially relevant in adolescents with MDD, who exhibit heightened suggestibility, variability in neurotransmitter system maturation, and developmental or gender-related differences [44]. Furthermore, gender-related differences in antidepressant response to intravenous ketamine treatment for adult patients with depression have been investigated, but with inconsistent findings. For example, Freeman et al. [45] found no significant difference in antidepressant response to intravenous ketamine infusions in females suffering from TRD, as compared with males with this diagnosis. However, a systematic review and meta-analysis found that males appeared to have slightly longer antidepressant responses to a single infusion of ketamine than females [46]. These factors uncover the complexity of comparing the efficacy of intravenous ketamine/esketamine with intravenous midazolam in treating MDD and highlight the need for careful study design and consideration of patient-specific variables in future research.

This systematic review incorporated a single RCT [25] that assessed the anxiolytic properties of intravenous ketamine in adolescents with MDD, demonstrating no marked benefit versus intravenous midazolam in diminishing anxiety symptoms. Anxious depression is prevalent among adolescents [47] and is linked to inferior treatment responses, diminished quality of life, and compromised well-being versus non-anxious depression [48]. Notably, unlike non-anxious depressed adults, individuals diagnosed with anxious depression exhibit diminished responses to intravenous ketamine regarding both antianhedonic [49] and antidepressant outcomes [50]. Likewise, recent research has demonstrated that intravenous esketamine exhibited a more marked anti-suicidal effect among adolescents diagnosed with non-anxious MDD versus their counterparts with anxious MDD [38]. Several studies have demonstrated that intravenous ketamine effectively reduces anxiety symptoms in adult patients with MDD [51,52], and it has shown an anxiolytic effect in treating refractory anxiety spectrum disorders [53]. Additionally, intravenous esketamine has exhibited an anxiolytic effect in adult patients with thyroid cancer [54]. Although Macejova et al. [25] reported no notable distinctions in anxiety symptoms between intravenous ketamine and intravenous midazolam, both treatments produced a substantial reduction in depressive and anxiety symptomatology in the short term. This effect may be attributed to the anxiolytic properties of midazolam [55].

This systematic review provides preliminary evidence, as no RCT has assessed the anti-suicidal efficacy of intravenous ketamine, and only one RCT [20] has evaluated that of intravenous esketamine, leaving the anti-suicidal effects of both agents inadequately established in adolescents with MDD. Although intravenous ketamine and esketamine demonstrate favorable tolerability profiles and have exhibited efficacy in ameliorating suicidal ideation and depressive symptoms among adult populations, their therapeutic effects in adolescent cohorts remain inadequately characterized. Suicide is a leading cause of death among adolescents, with recent studies showing an increasing trend in suicidal ideation and behavior [56]. A rapid reduction in suicidal ideation could markedly improve safety in acute-care settings, and intravenous esketamine appears to be a promising, albeit unvalidated, option for the rapid anti-suicidal treatment of adolescents with MDD. One plausible mechanism for this rapid effect involves a surge in brain-derived neurotrophic factor (BDNF). In adults with TRD, intravenous ketamine has been shown to markedly increase plasma BDNF levels within hours post-infusion [57,58]. Intravenous esketamine also appears to affect BDNF levels; for example, one study reported that a 0.25 mg/kg dose of intravenous esketamine markedly elevated plasma BDNF levels in patients with postpartum depression [59]. Although data directly from adolescents are lacking, emerging evidence suggests that BDNF dysregulation may serve an even more critical function in the pathophysiological mechanisms underlying suicidal behavior among adolescents versus adult populations [60], highlighting the therapeutic promise of intravenous ketamine/esketamine as a rapid-acting anti-suicidal intervention for adolescents diagnosed with MDD.

Only one RCT [20] in this review evaluated the neurocognitive effects of intravenous esketamine in adolescents with MDD, finding that intravenous esketamine markedly improved processing speed compared to midazolam, with no notable distinctions observed in other neurocognitive domains. In adults with MDD, intravenous ketamine/esketamine has been shown to improve neurocognitive function, with cognitive improvements observed independently of the antidepressant properties of intravenous ketamine [61]. A systematic review found that cognitive outcomes following ketamine or esketamine treatment for late-life depression were generally stable or improved, although long-term studies reported minor declines in reaction time [62]. Intravenous esketamine enhances cognitive function by modulating hippocampal and amygdalar subregional connectivity, particularly increasing functional coupling with the middle temporal gyrus and default mode network, thereby restoring neural flexibility and cognitive processing efficiency [63,64]. Despite these insights, the impact of intravenous ketamine/esketamine on neurocognitive function and its underlying mechanisms in adolescents with MDD remains to be fully elucidated through further research using standardized cognitive assessment tools, such as the MCCB [32,33] or the Repeatable Battery for the Assessment of Neuropsychological Status [65].

This systematic review identified nausea and dissociation as the most prevalent adverse effects of intravenous ketamine/esketamine compared to midazolam in adolescents diagnosed with MDD, while rates of other adverse events were similar across studies. Although discontinuation rates were generally comparable, Macejova et al. [25] reported a markedly higher dropout rate in the ketamine group (22.9% versus 0%). Despite this, intravenous ketamine/esketamine was generally well-tolerated in adolescents with MDD [20,21]. Furthermore, ketamine/esketamine have demonstrated safety and tolerability in adult [66] and elderly [67] populations with MDD. Nevertheless, certain patients may experience discomfort or distress due to the psychopathological and dissociative symptoms linked to intravenous ketamine/esketamine administration [68]. Although these effects generally exhibit self-limitation and disappear spontaneously without requiring intervention, careful medical observation remains crucial throughout the administration period. Notably, patients with a history of substance abuse may be more prone to drug cravings due to changes in reward circuits [69]. Therefore, intravenous ketamine/esketamine should be avoided in such individuals. However, only one of the three RCTs systematically reported safety data, highlighting the need for more thorough safety assessments of intravenous ketamine/esketamine in adolescent depression treatment.

### Limitations

This systematic review presents several limitations. First, conducting a meta-analysis was not possible due to considerable heterogeneity among the included RCTs. Second, the review encompassed only three RCTs with a limited sample size (n = 134, ranging from 17 to 63 adolescents with MDD), requiring careful interpretation of the results. Third, the three RCTs primarily investigated intravenous ketamine/esketamine in adolescents with MDD, among whom 92.5% were female, limiting the applicability of these findings to more diverse populations. Fourth, formal registration was not obtained for this review. Fifth, although the study by Dwyer et al. [21] received critical comments on PubPeer, it remained in normal publication status at the time of our review. In accordance with Cochrane systematic review inclusion criteria, we retained it. Even if this study were excluded from the analysis, the overall conclusions of the systematic review would remain unchanged. Finally, a formal inter-rater reliability assessment (e.g., kappa statistics) was not conducted during study selection, data extraction, and quality appraisal. While any discrepancies were resolved through consultation with a senior author, the absence of formal reliability testing may limit the reproducibility and consistency of the screening process.

## 5. Conclusions

Despite some encouraging findings, given the limited number, small sample sizes, and methodological heterogeneity of available RCTs, the current evidence base remains preliminary and insufficient to support definitive conclusions regarding the efficacy and safety of intravenous ketamine/esketamine in adolescents with MDD. Therefore, future adequately powered RCTs with comprehensive safety monitoring are strongly warranted to clarify its efficacy and safety profile in adolescents with MDD.

## Data Availability

All the data used in this manuscript have been included in the tables and figures.
