# Supplementary material for: Intravenous Ketamine or Esketamine in Adolescents With Major Depressive Disorder: A Systematic Review
Source: Alpha Psychiatry. 2026 Aug 27;27(5):48866. doi: 10.31083/AP48866 (PMC13731980; doi:10.31083/AP48866)
Supplement: Supplementary file 1 [file 2757-8038-27-5-48866-s1.zip › Supplementary Table.docx]

**Supplementary Table 1. Intravenous ketamine and esketamine in adolescents with MDD: adverse dissociative and psychopathological symptoms.**

| **Studies** | **Assessment scales** | **Findings** |
| --- | --- | --- |
| **Adverse dissociative symptoms** | | |
| Dwyer et al., 2021 (USA) | CADSS | Significant dissociative symptoms were observed with ketamine treatment compared to midazolam at the 1-hour time point, but not at other time points post-infusion. |
| Macejova et al., 2024 (Slovakia) | NR | NR |
| Zhou et al., 2023 (China) | CADSS | Significant dissociative symptoms were observed with esketamine treatment compared to midazolam at 30 minutes after each esketamine infusion, but not at 24 hours post-infusion. |
| **Adverse psychopathological symptoms** | | |
| Dwyer et al., 2021 (USA) | NR | NR |
| Macejova et al., 2024 (Slovakia) | NR | NR |
| Zhou et al., 2023 (China) | BPRS-5 | Esketamine group demonstrates a significantly higher the BPRS-5 score compared to midazolam group on days 1 and 3, but not at other time points. |
| Abbreviations: BPRS-5=5 items of the Brief Psychiatric Rating Scale; CADSS=Clinician-Administered Dissociative States Scale; MDD=major depressive disorder; NR=not reported. | | |

**Supplementary Table 2. Intravenous ketamine and esketamine in adolescents with MDD: discontinuation rate and adverse events.**

| **Study** | **Discontinuation rate (n, %)** | **Intravenous ketamine/esketamine group (n, %)** | **Intravenous midazolam group (n, %)** | **Findings^a^** |
| --- | --- | --- | --- | --- |
| Dwyer et al., 2021 (USA) | 0 (0) | 0 (0) | 0(0) | NS |
| Macejova et al., 2024 (Slovakia) | 8 (12.7) | 8 (22.9) | 0 (0) | NR |
| Zhou et al., 2023 (China) | 5 (9.3) | 2 (7.4) | 3 (11.1) | NR |
| **Study** | **Adverse effects** | **Intravenous ketamine/esketamine group (n, %)** | **Intravenous midazolam group (n, %)** | **Findings^a^** |
| Dwyer et al., 2021 (USA) | NR | NR | NR | NA |
| Macejova et al., 2024 (Slovakia) | NR | NR | NR | NA |
| Zhou et al., 2023 (China) | Nausea | 15 (55.6) | 7 (25.9) | P < 0.05 |
|  | Dissociation | 15 (55.6) | 3 (11.1) | P < 0.05 |
|  | Dry mouth | 8 (29.6) | 4 (14.8) | NS |
|  | Heart rate increase | 6 (22.2) | 1 (3.7) | NS |
|  | Sedation | 5 (18.5) | 8 (29.6) | NS |
|  | Headache | 5 (18.5) | 7 (25.9) | NS |
|  | Dizziness | 5 (18.5) | 2 (7.4) | NS |
|  | Somnolence | 5 (18.5) | 0 (0) | NS |
|  | Blood pressure increase | 4 (14.8) | 2 (7.4) | NS |
|  | Agitated | 4 (14.8) | 0 (0) | NS |
|  | Hallucination | 3 (11.1) | 0 (0) | NS |
|  | Paresthesia | 3 (11.1) | 1 (3.7) | NS |
|  | Tremors | 2 (7.4) | 1 (3.7) | NS |
|  | Anxiety | 2 (7.4) | 0 (0) | NS |
|  | Agitation | 1 (3.7) | 2 (7.4) | NS |
|  | Stomachache | 1 (3.7) | 1 (3.7) | NS |
|  | Vision blurred | 1 (3.7) | 1 (3.7) | NS |
|  | Suicidal behavior | 0 (0) | 1 (3.7) | NS |
| ^a^ The differences between ketamine/esketamine groups and midazolam groups at the treatment endpoints.  Abbreviations: MDD=major depressive disorder; NA=not applicable; NR=not reported; NS=not significant. | | | | |
